# Editorial: Embryonic, reprogrammed, and multipotent cells in domestic animals: *In vivo* and *in vitro* mechanisms and applications

**DOI:** 10.3389/fvets.2023.1151065

**Published:** 2023-02-14

**Authors:** Fabiana Fernandes Bressan, Carlos Eduardo Ambrósio, Tiziana A. Brevini

**Affiliations:** ^1^Department of Veterinary Medicine, Faculty of Animal Science and Food Engineering, University of São Paulo, São Paulo, Brazil; ^2^Department of Health, Animal Science and Food Safety and Center for Stem Cell Research, Università degli Studi di Milano, Milan, Italy

**Keywords:** regenerative medicine, stem cells, induced pluripotent stem cells, mesenchymal stem cells, stem cells niche, translational medicine

Stem cells are a fascinating tool for regenerative medicine, mainly due to their potential to repair damaged tissue and model diseases and syndromes *in vitro*. They are commonly classified according to their stage of differentiation, which is disclosed mainly by their epigenetic profile and origin. This special edition, therefore, aims to bring innovative and applied usage for multipotent or pluripotent cells and to discuss mechanisms, applications, and the consolidation of new technologies, using stem cells or derived products, focusing on domestic animals.

In this context, adult stem cells are already well-studied and characterized as multipotent cells and are already widely used in regenerative medicine, primarily as immunomodulators (1). Pre-clinical and clinical trials using these cells are emerging in veterinary medicine; however, they still lack robust protocols, particularly when compared to human equivalents. In this Research Topic, data from Peyrecave-Capo et al. assessed the safety and clinical feasibility of the therapeutic use of umbilical cord serum (UCS) eye drops in cases of spontaneous complex ulcers in horses. UCS and human autologous serum were compared regarding cytokine and growth factor profiles, and the results contribute to the generation of an adjunctive therapy for equine complex non-healing ulcers.

Pluripotent stem cells, on the other hand, are mostly studied and characterized in human and mouse models rather than in other species, despite their importance for their potential use in many aspects of regenerative medicine as well as for the understanding of the initial development of mammals. The derivation and maintenance of embryo-derived pluripotent stem cells (ES) in domestic species is still extremely challenging and presents inconsistent results regarding the preservation of *in vitro* pluripotency, despite recent livestock advances (2–4). In this context, the derivation of *in vitro* induced pluripotent cells (iPS cells or iPSCs) has shown encouraging insights into domestic species due to its differentiation into various cell types and, in special, due to its ease of obtention from adult tissues and advantages of having a known genetic background. Nonetheless, it still lacks reproducible reprogramming and adequate niches and conditions for adequate and safe fate induction (5).

In this Research Topic, two articles reported remarkable results on the use of reprogrammed cells, comprising both the optimization and safety of the protocols and also reproducible differentiation, aiming for medical use of these cells. Chakritbudsabong et al. reported the generation of porcine induced neural stem cells using the Sendai virus, an integration-free methodology that does not alter the genetics of the host cell and therefore comprises a safer and more adequate protocol targeting further biomedical use. They have also successfully established porcine iNSCs (piNSCs) that expressed NSC-specific proteins and were able to develop into neurons and glial cells (Chakritbudsabong et al.). Chandrasekaran et al. also reported the generation of neural derivates using iPSCs; however, from an elderly dog presenting mild cognitive impairment, a great achievement for further disease modeling *in vitro*. These results provide both new perspectives on iPSC use in veterinary medicine and also offers a conveniently accessible large animal model for assessing the efficacy and safety of transplantation in human medicine.

Finally, the importance of the stem cell niche and its regulatory mechanisms was assessed by Arcuri et al. through the generation of trophoblast-like cells from hypomethylated porcine adult dermal fibroblasts. This report brings significant advances in regenerative medicine by supporting viable conditions and models that modulate phenotypes for use in both basic research and applied medicine, such as to characterize embryo implantation mechanisms better or to model developmental disorders based on trophectoderm defects (Arcuri et al.).

In summary, the present Research Topic and all the studies mentioned above bring together both advances and perspectives on how to successfully overcome the main and fundamental barriers related to the translation of protocols and results to the veterinary field. In addition, the data collected present solid evidence for the use of differentiation protocols and pre-clinical assays. Such achievements may support the prominent development of biotechnologies, mainly concerning the establishment of cell lines, robust differentiation protocols, adequate *in vitro* culture environments, and pre-clinical assessments.

## Author contributions

All authors contributed to the article and approved the submitted version.
